# Changing Perspectives of Local Therapists Eight Years after the Implementation of an Occupational Therapy Service in a Unique Himalayan Cross-Cultural Setting

**DOI:** 10.1155/2021/5520195

**Published:** 2021-09-18

**Authors:** Gaby Scheidegger, Zhang Ting Ting, Caroline Bastiaenen, Michael Nagler

**Affiliations:** ^1^ErgoService, Aarberg, Switzerland; ^2^Chinserve, Aarberg, Switzerland; ^3^Department of Epidemiology, Maastricht University, Maastricht, Netherlands; ^4^University Institute of Clinical Chemistry, Inselspital, Bern University Hospital, and University of Bern, Bern, Switzerland

## Abstract

*Background*. Whether concepts and principles of Occupational Therapy (OT) can successfully be applied to non-Western and cross-cultural settings is being intensively discussed. *Aims/Objectives*. We explored the changing perspectives of local occupational therapists (OTs) eight years after the implementation of an OT service in a Himalayan cross-cultural setting in terms of (1) treatment applied, (2) professional identity, and (3) cross-cultural interactions. *Material and Methods*. A qualitative study design was chosen, and semistructured interviews were conducted in all employed practitioners (a) during implementation and (b) eight years later (*n* = 7). Questions were carefully formulated in order to narrow down the intended issues but respecting crosscultural differences. The framework method was implemented for data analysis. *Findings*. Long-term empowering local OTs resulted in the successful development of a sustainable OT department in a unique Himalayan cross-cultural setting. Practitioners became aware of their therapeutic potentials, a clear sense of professional identity was developed, and it was recognised that sensitive cross-cultural practice is only achieved by an ongoing and intentional cultural learning process. *Conclusions and Significance*. Our findings suggest that OT can be applied to non-Western cross-cultural settings.

## 1. Introduction

It is a matter of debate, whether concepts and principles of Occupational Therapy (OT) can be applied to non-Western and cross-cultural settings. The discussion expanded around the year 2000, as some authors argued that OT theory and practice, which have been developed in the United States, are heavily influenced and value-laden by Western worldviews and lifestyles [1–5]. The search for an OT practice relevant in a global perspective stimulated an intensive discussion about aspects of cultural competences and sensitive cross-cultural practice [6–14]. Developing cultural competences is a lifelong journey and a process of ongoing personal growth [9, 15–17]. Essential to cultural competences is (a) reflecting about one's own culture and that of others (*knowing*), (b) engaging in multicultural contexts (*doing*), and (c) developing an understanding of others who are different from oneself (*becoming*) [9, 18–22]. Accordingly, OT is a holistic approach, which is concerned with the daily life of its clients, individually, as well as within their social and work context [23]. Occupational therapists (OTs) work and interact with their clients in their natural everyday life. Therefore, it is essential to observe how people live their lives in their environment, how they value things, and how they take into account the client's worldview and culture [9]. Following these considerations, OTs are in an excellent position to gain specific first-hand knowledge and understanding of the needs and interests of those in need within their specific culture. Reviewing the listed arguments, OT has a predestined position to make a valuable contribution to global health [23]. The World Federation of Occupational Therapists (WFOT) aims to enable all people, regardless of culture, ethnicity and social background, to be engaged in occupations that support their well-being and the well-being of their communities [1]. To support the development of OT in non-Western settings, researchers have been requested to study long-term experiences of OTs working in different cultural settings [24–27].

In China, OT is a relatively new health profession. The Chinese healthcare system has been evolving by embracing both traditional Chinese and Western medicine content [28]. Chinese rehabilitation medicine is influenced by both theories [29, 30]. Contemporary rehabilitation medicine, which did not include OT, began being practiced in the early 1980s [29, 31, 32]. The Chinese government started to recognise the need for rehabilitation services around the year 2002 [28, 29, 31, 33]. A growing number of people with injuries and disabilities could not be treated and were not available as a workforce [31, 32]. In 2010, the number of people with disabilities in China was estimated to be 85 million (about 6% of the population) [31–33]. This situation was aggravated by demographic changes resulting from birth control, longer life expectancy [32], and rapid social, economic, and institutional transformations [28, 34, 35]. Even contributing more, severe earthquakes (Sichuan 2008, Yushu 2010) left many hundred thousand or even millions injured [36, 37].

At that moment in time, first efforts to implement rehabilitation services started, primarily using traditional Chinese techniques. Besides, government-sponsored health insurance was introduced even to remote areas [28, 31, 38]. Starting from institutions such as Hong Kong Polytechnic University [39], the first OT departments were implemented at the China Rehabilitation Research Centre [40] and Medical University Kunming [31]. These initiatives were supported by foreign OT experts who held training sessions in a few hospitals [41]. Even the national media (CCTV) mentioned OT services by covering rehabilitation stories and pictures [42]. Not until 2018, China was accredited as a member by the WFOT and OT was recognised as a profession by the Chinese government [43]. Despite all these efforts, OT services are still lacking in most Chinese hospitals [29, 33, 39, 44].

Why is it challenging to establish OT services in the Chinese healthcare system? First and most important, crucial knowledge about OT is essentially lacking. Next, training facilities are minimal [31, 39, 45]. An essential condition as a structured education programme only is available in very few institutions [29, 31]. Therefore, most OTs received training as apprentices and not in a structured programme [31]. Only a few textbooks exist within Chinese language, which are often outdated and not evidence-based [31, 40]. Many OTs are rehabilitation practitioners and learned the job by training after examples that were limited in skills and number of OT techniques [31]. Secondly, the professional identity of being an OT is practically unknown [39, 44]. OT does not receive appropriate attention from society because the concept does not resonate with the Chinese understanding of healing [45]. Thus, explaining the effects of OT to patients and other healthcare professionals is difficult [44]. Thirdly, OT takes place often in a cross-cultural context [14, 44]. OT has been developed in the Western worldview and is based on the assumption that patients' independence is the essential aim [1]. In contrast, interdependency and roles in communities are much highly rated in Asian and Chinese societies [3, 14, 39, 46]. Activating techniques of OT are in contrast to traditional Chinese beliefs of being passive in order to get healed from an injury or disease [32, 45]. Older patients reject instructions from younger OT personnel because older people expect to be honoured and respected and asked for guidance [2, 47]. In addition, younger OT personnel fear “losing face” following a confrontation with the patients [47–49].

Language barriers between OT and patients play an additional role in areas with minorities [44, 50]. Satisfactory communication may be influenced by language and cultural differences between therapist and client [50]. Overmore, patients with little formal education might not understand the instructions nor the potential benefits of OT [44]. In remote areas, the proportion of people with illiteracy is still high [37, 38].

Qinghai is a Himalayan province of China with a unique ethnical structure of the population [51, 52]. It is socially and culturally extremely diverse due to different groups of Tibetan (Amdo, Kham), Muslims (Hui, Salar), and Han (Chinese) [51–54]. Even within these ethnic groups, several different communities with particular languages, beliefs, and customs do exist [51–56]. Longstanding conflicts among these groups are recently aggravated by the development strategy of the Chinese government, which will transform the area from a nomadic agricultural society to a 21st century technology within a few years [52–55]. Due to various programs, a large population of Han Chinese has resettled to Qinghai in order to develop the economy [52, 55]. In addition, the ecological migration policy of the Chinese government forces Tibetan pastoralists from their nomadic livelihood into new resettlement villages [57, 58]. Within these villages, people suffer from various diseases due to lacking sanitation and running water, as well as poverty due to missing jobs. In rural nomadic areas of Qinghai, women's access to education was low (in 2004) at 15% (*n* = 59/399) for any formal schooling; adult female literacy was <20% (only 2% reading easily and 20% reading with difficulty) [37]. In 2018, the illiteracy rate in all over Qinghai still is reported as 15.1% of the population [59]. As a result of all these factors, Qinghai is regarded as one of the poorest provinces in China [55]. Compared to the rest of China, access to healthcare is regarded as inferior [37, 60]. As an example, skilled maternal care and Caesarean delivery are virtually inaccessible in rural areas of Qinghai province [37, 60]. Up until 2009, OT even was not existent in Qinghai province.

Recognising high numbers of untreated patients with injuries and disabilities, the first assessments of needs were conducted by foreign experts (Chinserve relief and development [61]) in 2008. Supported by the head of the hospital, an OT and Physiotherapy (PT) Department was planned and gradually developed at Qinghai Red Cross Hospital (QHRCH [62]), a leading tertiary hospital in Xining, the provincial capital of Qinghai province [44]. A foreign OT expert (GS, first author) was employed as the head of the department between 2009 and 2015. From the start, detailed concepts for training and day-to-day duties and responsibilities were implemented, and content for teaching was defined. Regular teaching sessions were organised within the department as well as together with foreign experts in OT and PT. Further development of the department was organised as a continuous sequence of learning, working (application), evaluation, and teaching [44]. The number of OT and PT practitioners was gradually increased up to 12, and the department was turned over to a local OT head in 2015. At the beginning of the service in 2009, several key issues were identified by the local OT team [44]. First, professional service must be developed to provide treatment options open to everybody by training skills and establishing knowledge (treatment applied). Second, as OT is a new treatment and profession, practitioners have to fully understand the concept and explain it adequately to other medical professionals as well as to the patients (professional identity). Third, patients originate from several nationalities with a very different cultural background. Cultural and language barriers exist that prevent adequate and equal treatment for all patients (cross-cultural interactions) and should be taken into account on a day-to-day basis.

### 1.1. Aim

The present study is aimed at observing the changing perspectives of local OTs eight years after implementing an OT service in a Himalayan cross-cultural setting concerning (1) treatment applied, (2) professional identity, and (3) cross-cultural interactions. We aimed to answer the following research question: how did local OT's experience and make meaning of their cross-cultural therapeutic encounters, and how did this change over time with regard to their professional identity and position within the department?

## 2. Materials and Methods

### 2.1. Study Design and Setting

To answer the research question mentioned above by observing the subjective perspectives and cultural awareness, we chose a qualitative study design, with a small number of participants [63–65]. Semistructured interviews were conducted in the same study population at two time-points: (1) during implementation of an OT department [44] and (2) eight years after implementation (reported in this manuscript). The findings of both interviews were compared in order to analyse changes over time in perspectives and cultural awareness.

The study was performed at the OT department of QHRCH, a leading tertiary hospital in Xining, the provincial capital of Qinghai province.

### 2.2. Interview Design

A qualitative design with semistructured interviews was performed at both time points in order to collect scientific sound data on OTs views and experiences [66, 67]. In the development phase of the study, unstructured interviews preceded the semistructured interviews in informal settings followed by a pilot interview in order to develop a comprehensive interview guide and protocol [44]. The questions of both interviews were identical; the interview guide is reported in Table 1. The first interviews were conducted orally, and voice was recorded. Drawing from the experiences of the first interviews, we decided to use a written questionnaire for the second interviews. This more anonymous procedure gives the participants the chance to reply openly, without any fear of “losing face” or giving the “wrong answer” [68]. Their statements, therefore, are expected to be more reflected and less spontaneous, and the participants have more time to think about their answer. The questions were carefully formulated in order to narrow the intended issues in a circular manner and avoiding cross-cultural offending behaviour (linguistic sensitivity (66)). Introducing questions were followed by follow-up questions, probing questions, specifying questions, interpreting questions, and debriefing questions [67]. At least two different questions on the same issue were included, in order to confirm the individual meanings. Translation of the questions (from English to Chinese) has been done by the same research assistant at both time-points.

### 2.3. Participants and Data Collection

Participants were selected following a purposive sampling design on the base of their work engagement with the OT department [65, 69]. The inclusion criteria were (a) Chinese degree in rehabilitation medicine, (b) those currently employed at the OT department of the QHRCH hospital, (c) and those working as an OT practitioner. As an exclusion criterion, practitioners working at the rehabilitation department who are not trained within the OT curriculum were not considered for participation (Table 2). Extensive information about the aim and intent of the study was prepared beforehand and provided in a particular meeting several days before conducting the interviews. Plenty of time was provided for questions about the procedures [70]. Informed consent forms were prepared, translated (English to Chinese), and explained in detail. All participants gave written informed consent before the interviews and oral consent after the interviews. A comprehensively trained local research assistant conducted oral interviews (first-time point) to support an honest response, without any fear of “losing face” or giving the “wrong answer.” As a native Chinese speaker, the research assistant was quickly able to keep the conversation going and ask in-depth and follow-up questions. The research assistant was selected as a member of the same cultural background and age, however not working at the same hospital. Sufficient time slots were reserved to facilitate honest answers. Written interviews were conducted at the second time-point. Translations were refined and annotated, aiming to represent as closely as possible the meaning of the individuals.

### 2.4. Research Team

In order to meet the study aims and achieve trustworthiness and credibility, we build a multicultural, multilingual and multidisciplinary research team [44, 65, 71, 72]. This collaborative approach is increasingly used in cross-cultural research projects [72, 73]. The research team included four members. The first author and principal investigator is an experienced Swiss OT who lived in Qinghai province for more than 18 years and conducted several related research projects [25, 44]. A Chinese research assistant was selected with the same cultural background and similar age as the participants and having sufficient skills in English speaking and writing fluently (English teacher), however working at an independent institution. A Dutch epidemiologist, with a broad experience in quantitative, qualitative, and mixed method research designs and methods supported the writing process. An experienced principal investigator in a variety of health-related research projects supported the design, interpretation, and presentation of the project [74–77].

### 2.5. Analysis

The framework method used was similar to content analysis to investigate the data [63, 71, 78]. Framework analysis offers a systematic, flexible, and comprehensible approach for the thematic analysis of interview transcripts using a deductive-inductive method [71]. This method is increasingly popular in health research. The process was guided and discussed with an external supervisor. All oral and written data were translated and transcribed by the research assistant and then compared and agreed with the first author. Translations were refined and annotated, aiming to represent as closely as possible the meaning of the informants. In the first familiarisation step, the original Chinese texts and English transcripts were reviewed in full-length several times (familiarisation). In the second step, a thematic framework was developed by listing the key ideas, concepts, and recurrent themes (framework building). This step was discussed and annotated with the academic supervisor. The dataset was indexed and coded, respectively (indexing), drawing from the thematic framework. Then, data were rearranged according to the different themes of the thematic framework (charting). To conduct member checking, the first author visited the department in 2019 and discussed and checked the analytical process outcomes with the participants. Finally, the critical characteristics of the data, as laid out in the charts, were mapped and interpreted in order to provide a schematic diagram of the phenomenon (mapping and interpreting). The first author engaged in a peer-debriefed reflexive process with the fourth author in order to examine personal perspectives and biases (66). We focused on retaining the original meanings of the interviewees. This mapping and interpretation were influenced by the research question, but emerged themes were taken up. With this, the analytic process started deductively from a specific research question but inductively included emerging ideas [79].

### 2.6. Ethical Considerations

This study was conducted in accordance with the declaration of Helsinki [80]. Participants were comprehensively informed about the aims, methods, and intent of the study several days before conducting the interviews, and time was provided for questions [70]. It was particularly mentioned that participation is completely voluntary and that the local OTs have the right to withdraw consent at any time. Also, they were told that participation, as well as nonparticipation, does not influence their professional activity or position. Informed consent was sought using translated forms (in Chinese), which were explained in detail. All participants gave written informed consent before the interviews, and additional oral consent was collected again after finishing the interviews. Before analysis, all interview transcripts were coded. No regulations and formal ethical committee existed in Qinghai province. Hence, the protocol was approved by the general medical director of QHRCH. The protocol was additionally approved by the Research and Ethics Governance Committee of the University of Brighton, UK [44].

## 3. Findings During Implementation and Eight Years Later

Three broad themes emerged and are presented below. The first theme reveals the perception of the participants regarding OT practice. The second theme represents the understanding of their professional identity, and the third theme constitutes the reflections on their cross-cultural interactions. These themes were brought together across all interviews, with efforts to retain the language (Chinese to English) of the respondents as close as possible to “original” [63, 70].

**Figure 1:**
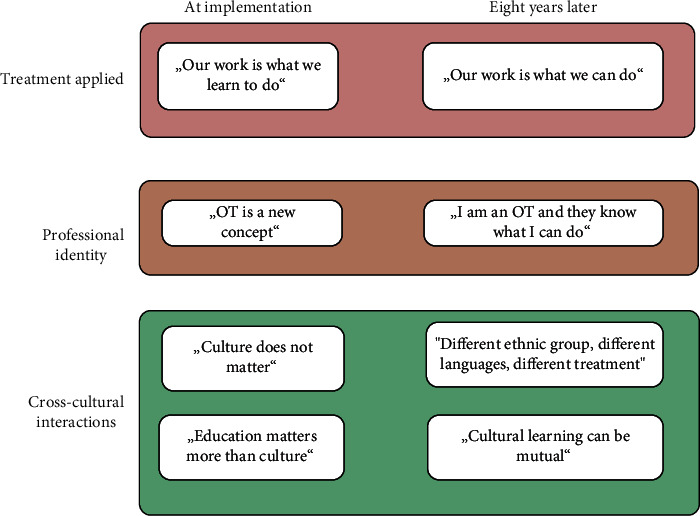
Changing perspectives of local therapists after implementation of an occupational therapy service in a unique Himalayan cross-cultural setting.

An overview is given in Figure 1.

### 3.1. Treatment Applied

#### 3.1.1. What Does OT Mean?

During implementation, most participants found OT a new concept, and they were aware that their knowledge is limited. The OT practitioners realized that neither patients nor medical professionals are aware of what OT is. The participants described OT as the profession they currently study.

“OT is new. Most people do not know about the possibilities of therapy.”

“Rehabilitation and OT just developed in recent years.”

“I did not know about OT and rehabilitation myself when I started medical school.”

“Our work is what we learn to do.”

Eight years later, the informants perceived OT as the profession they can practice, and they explained the professional contents with confidence.

“I work as an OT.”

“OT means to treat patients, help patients, to recover to good health.”

“OT is to dig out and use the best potential of our body's function.”

“To accomplish basic living, to be independent, and being confident.”

“Helping them to live physically independent and participate in social activities.”

“Our possibilities become better and better, there are a wider variety of courses, and we can participate in local and national courses.”

#### 3.1.2. Treatment Process

During implementation, most local therapists had a basic understanding of a treatment process. They were aware that there are different phases of treatment: an assessment, a treatment plan, and an intervention. They started to use treatment protocols in daily practice.

“We check the clients first and make an assessment, then we make a treatment plan, then we start to train/exercise the clients every day, and after the treatment, we write the process down and keep a track.”

Eight years later, the interviewees specified the contents and parts of the treatment process clearer and more in detail. They addressed the different stages of treatment with raised awareness and focus. Ongoing evaluation of the treatment, continuous dialogue with clients and relatives, and evidence-based practice were considered important aspects of the treatment process.

“When I see a new patient, I look at the diagnose … make the plan how to treat and about the treatment direction…, then I check and make a specific evaluation,… adapt the treatment according to the situation.”

“Speaking of an evaluation, set goals, treatment plan, and implementation into practice.”

“Evidence-based practice (is to have lots of scientific studies to make treatment more efficient), makes me find better ways to treat the patients.”

“We communicate with the patients, understanding them, and their future health.”

#### 3.1.3. OT Clients and Interventions

During implementation, the local therapists spoke about patients with orthopaedic or neurological conditions as their potential clients. They also articulated some specific treatment and intervention modalities.

“We treat patients who have paraplegia, hemiplegia, and there are children with cerebral palsy. There are also patients after bone fractures, as well as with depression.”

“We use active movements and passive movements, we adapt casts…”

“In the case of a hemiplegia, we treat patients on the bed or beside the bed, do some passive activities, then teach them to do active exercises; further we add balance, sitting and standing balance, as well as physical coordination.”

“We help them to overcome limitations by applying adaptations like walking aids and exercise with them ‘activities of daily living.”

Eight years later, the answers of the local OTs indicated increased specialised knowledge and understanding. Several treatment modalities were described, and sound details were provided. In addition, stories of success were shared, and clients were approached more holistically.

“Stimulation of life, fun and family environment.”

“Supporting the client's recovery of the hand- and arm functions, specific movements, as well as daily life abilities...”

“To help people who need therapy to accomplish basic living, being independent, and being confident.”

“To help patients to regain and stimulate sensibility.

“We provide task-oriented training, muscle exercises, hand-function training, balance exercises, mirror training, and ADL-training.”

“A patient who had a bone fracture because of a car accident,… came to our department, after three months of treatment (in another place),… his knee got normal, and he is able to walk again.”

“There are many clients with hemiplegia who learn to walk again and regain many ADL abilities.”

#### 3.1.4. OT Aims and Therapeutic Concepts

During implementation, the participants reckoned the professional tasks of OT as to approach the patients and their families with a focus on “activities of daily life” (ADL) and a concept to teach skills for a more independent living.

“The most important aspect of ADL training is for the client to go back to the family and manage to eat and get dressed by themselves.”

“If there is no one (at home) to take care of the patient, for them to be able to move and turn in the bed, and move from the bed to the bathroom by themselves is most important. The main aim of these clients is to manage at home.”

Eight years later, the local OTs expressed therapeutic aims and concepts more in-depth. Awareness grew that clients shall participate actively in OT and that functions can be restored over a period of time. Independence and participation were understood as core values of OT and implemented in a justified way.

“Participate in society, improve life abilities, being independent, being confident,…”

“That they can normally live in family and society.”

“Supporting the clients to regain body function and life abilities as well as helping them to recover, and to restore functions.”

“It is an active participation of the client.”

“Rehabilitation is a change, which needs the active involvement of the client and is a process of recovery.”

“To minimise disability and support active participation.” “To help people who need therapy to accomplish basic living, being independent, and being confident.”

### 3.2. Professional Identity

#### 3.2.1. Role as OT

During implementation, the participants reflected that they have only a little imagination, what OT is and what an OT can do. At that time, OT was an unknown and new profession in Western China and Qinghai province.

“I did not know about rehabilitation myself when I started medical school.”

“Before I studied, I saw children with disabilities on the street, and I did not realise that their situation could be improved… after I studied, I started to realise more.”

“It was a new profession and a new approach.”

“Rehabilitation has just developed in recent years.”

Eight years later, the informants claimed for themselves to perform as OT professionals. Meanwhile, they were able to participate in national and international courses, and by now, all local OTs graduated with a medical BSc degree.

“I am an OT, and I know what I can do.”

“I understand my OT work, and I have studied it.”

“We need to understand medical science and study a lot of medical content.”

#### 3.2.2. Role within the Hospital

During implementation, the local therapists' understanding about their professional characteristics and role within the hospital was unclear and sparse.

“The doctors give surgery, and the nurses give injections; we finish the middle process.”

Eight years later, the local OTs realised that doctors and patients value the potentials of OT increasingly. They appreciated that there are more clients referred to the OT department from other health departments.

“Doctors and patients know about OT and are more and more aware of OT.”

“The OT department is gradually more important within the hospital.”

“The doctors value our department increasingly.”

“Our position as a department has improved a lot compared to before…”

“The hospital values our department more and more.”

“Other departments and other medical workers introduce clients to us.”

“The children's department support our work a lot and appreciate us.”

“Medical workers in the hospital know about us and introduce us to the clients.”

“We are equal with doctors and nurses.”

#### 3.2.3. Role within the Society

During implementation, the participants reflected that they need to explain the possibilities of OT to family and friends as well as to clients.

“OT is new. Most people do not know about the possibilities of therapy.”

“OT was new to clients…”

“Knowledge was passed on by word of mouth and television; they hear from friends, other clients, advertisements.”

“Clients are often not willing to pay for treatment.”

Eight years later, the interviewees confirmed that people know what OT is and that the clients are willing to pay for OT. An increasing number of patients now had health insurance.

“People know about OT.”

“OT is introduced at the television.”

“From TV, when they stay in the hospital,… from other people's introduction.”

“Clients are willing to pay for the treatment, and people have now health insurance.”

“Most patients are willing to pay, especially if they are having a good family condition (if they are rich).”

### 3.3. Cross-Cultural Interactions

#### 3.3.1. Cultural Differences

During implementation, the local therapists stated that they interact with patients from different nationalities but “treat” everyone the same, and the cultural background does not affect the treatment. They considered other aspects more important than culture.

“There are Tibetan, Hui and Han patients.”

“No matter, Tibetan, Hui or Chinese, they are all the same.”

“We treat them all the same way; their cultural background does not influence the treatment.”

“I think patients' nationality and culture does not influence my therapy, and it does not influence how I treat the patient.”

“Culture does not matter – patients are all the same (are all human).”

“We do not adjust treatment for different cultures. We adapt because of different interests and hobbies.”

“We Han Chinese are sometimes unaware of the different cultures; we speak words that could hurt them,… this we should avoid.”

“No matter whether you are a Han Chinese or from a minority group or even from a nomad place, if they come, they all want to cooperate with the treatment.”

“Patients are all the same – the influence of culture is not so big, and they are not influenced greatly by their cultural background.”

Eight years later, The local OTs described what culture means for them; they had their definition of culture. They confirmed clients with different ethnical background and adapted treatment accordingly.

“Culture is a combination of history, place and time.”

“Culture is knowledge, cognition, science and technology, ability, custom, and language.”

“Culture consists of education, ethnic culture, cultural custom, humanistic culture and social/popular culture.”

“Culture consists of an ethnic group, different (ethnic) language, family background, religious belief, and education.”

“A person's upbringing and the way he/she does things, environment and people he/she has a relationship with.”

“I adapt my treatment because of different cultural background.”

“Cultural backgrounds are critical to therapy work. Different ethnic groups, different languages, different treatment.”

#### 3.3.2. Language Barrier

During implementation, the participants realised that language is a challenge and can be a barrier during treatment. Their communication with patients from different ethnic groups was limited; and sometimes, they used help for translation.

“Language is a barrier in communication, and sometimes we are not able to understand each other, we need translators.”

“There are some difficulties because of different languages.”

“I am not able to speak Tibetan; … sometimes they are not able to understand me, either. … We need to find someone for translation.”

“They have even different Tibetan dialects.”

“I learnt some simple words like ‘move the leg,' … ‘pull,' ‘stand,' ‘painful or not.'”

“Hui minorities,… normally speak Qinghai dialect (of Chinese). There are some dialects, such as Salar language and from other Islamic people groups,… they use some Arabic when they pray. I adjust my way of speaking.”

“If we use Qinghai dialect to say things, they feel closer,… and more connected.”

“Just to have professional knowledge is not enough, but learning some other languages is also important.”

Eight years later, the interviewees confirmed that the different languages were still a challenge. However, the OTs found ways of communication and adopted a variety of solutions among different languages.

“The only thing is sometimes I cannot understand the different languages, I do not know how to explain the therapy in a different language.”

“Different ethnic groups, different languages, different treatment.”

“There are translators: I ask a translator to help when I do not understand the language.”

“Yes, I adapt the treatment. For example, with Tibetan people, when I cannot understand the language, I use more body language.”

“Different cultures, different languages; therefore, different treatment.”

“I do not know some aspects of their culture, but try to communicate more to understand their culture and respect their beliefs.”

“Most ethnic groups now all know some Chinese; if they do not, we ask a translator or use body languages, they can speak some simple keywords.”

“I speak Chinese. Different ethnic groups have different languages; they communicate with each other and learn from each other.”

“I speak Chinese; I ask a translator to help when I do not understand the language.”

“They are humble in communication, and they respect each other. I speak a little bit of minority language.”

“I teach a Tibetan girl to speak and learn Chinese. I hope her situation can get better, not poor anymore.”

#### 3.3.3. Religion

During implementation, the local therapists were aware that there are different religions. However, mutual respect is more important. Some knowledge about the expressions of faith among the resident nationalities was present.

“Regardless of cultural background, we should respect each other.”

“We will not ask about their background; we will not set out to get to know their customs and beliefs initially. In the treatment process, if they mention it, we can talk about it, but if they do not mention it, we will not ask about it.”

“Tibetan people pray (publicly) with those prayer beads … everyone prays until the prayer beads are getting smaller a smaller, they pray a lot.”

“We do not have to adjust treatments for different cultures … these functional training will not affect their social customs; we really will not affect or influence their religion or rules.”

“I look at their interests, and I learn about the person's interests and hobbies.”

“Our Han culture is getting lazier and less connected, but the minorities, they feel like a big family; they are more united.”

“Minorities are very united. Their sense of unity is powerful.”

“If they are from the city, or the countryside, all in all, this does not have an influence, I always treat everyone the same.”

“Tibetans are very polite; even some educated people are not as polite as they are; I feel it is like this, maybe because of their nationality and belief.”

“There is an older and more modern expression of the Islamic religion. The new one is more open; the old one is traditional. … They go to the mosque to pray every day.”

Eight years later, the local OTs confirmed that religious belief is a significant part of the culture. They confirmed that it is essential to respect and understand a person's belief.

“Culture consists of an ethnic group, different language, family background, religious belief, and education.”

“I do not know some aspects of their culture, but try to communicate more to understand their culture and respect their beliefs.”

“There are some Tibetans who think their religion works better than treatment.”

#### 3.3.4. Education

During implementation, the participants stated that the level of education matters more than culture concerning the patient's receptiveness of therapy.

“The level of education matters more (how people are able to participate in treatment), than culture.”

“Level of education, educated or not, matters most. People who did not go to school are able to understand less.”

“If someone went to school, there is an understanding of curing a disease,… the patients then also have better co-operation with the doctor,… and they do what we tell them.”

“If they are not educated, they do not understand the meaning of moving or not moving … if they are highly educated, they can understand that to do this kind of treatment will be good for their recovery.”

“Tibetans are very polite; even some educated people are not as polite as they are; I feel it is like this, maybe because of their nationality and belief.”

“If they are from the city, or the countryside, all in all, this does not have an influence. I always treat everybody the same.”

“Patients coming from some areas in the countryside, are not so well educated and do not understand professional wording.”

Eight years later, the informants confirmed that education matters for the receptiveness of OT. They realised that cultural learning could be mutual.

“Culture consists of an ethnic group, different language, family background, religious belief, and education.”

“Cultural learning comes from education (from a young age on), and family life.”

“Different ethnic groups have different languages; they communicate with each other and learn from each other.”

“Any time any situation is to learn good things from others and make up my own weakness.”

## 4. Discussion

This research is the first investigation studying changing perspectives, as well as the professional development, of local OTs in a non-Western and cross-cultural setting over time. The aim of the present study was to observe (1) treatment applied, (2) professional identity, and (3) cross-cultural interactions. How did local OT's experiences make meaning of their cross-cultural therapeutic encounters, and how did this change over time, regarding their treatment, professional identity and position within the department, and their cross-cultural awareness and competences?

By empowering local rehabilitation therapists in a hands-on apprenticeship model, it was possible to successfully develop an OT department in a unique Himalayan cross-cultural setting [44]. The OT service helped to meet the urgent need for rehabilitation in China [31, 32, 39], that became visible after the vast earthquake in 2010. Outcomes of our project support the vision of the WFOT to “enable all people regardless of culture, ethnicity, or social background, to be engaged in occupations that support their well-being and the well-being of their communities” [23, 81].

### 4.1. From Unknown to Applied, Professional OT

Over the period of eight years, the local therapists learned from scratch, to practice and to understand a completely new type of profession and treatment [28, 29, 32, 82]. Eventually, the OTs were able to practice and explain professional contents with confidence. They shared specific stories of successful outcomes. The variety of treatment modalities increased, and concepts were understood more in-depth. OT practice became increasingly meaningful, and the therapists were able to name specific aims as “to minimise disability” and “to support active participation in society.” Besides, therapists recognised the perspectives and hopes they can raise in clients and their families regarding active participation in society. They became aware that OT intervention is a process of change, which supports recovery and needs an active involvement and participation of the client. These findings are supported by previous research conducted in different cross-cultural settings and emphasising the learning context and the meaning and motivation provided with the study curriculum [83, 84]. Accordingly, Eleyinde et al. described the increase of perceived relevance of the OT curriculum over time [85]. The importance of local context was also emphasised by the former president of the WFOT [86].

### 4.2. Development of Professional Identity and Role

Whereas the OTs were not aware of their professional identity during implementation (“I did not know”), they realised more and more what it means to be an OT (“I am an OT and they know what I can do”). The practitioners became aware of their professional skills and abilities and were able to explain this to clients, medical staff, friends, and family. The OTs perceived that they could contribute to society by caring for a large number of disabled individuals in China [31, 39]. They recognised the increasing awareness among hospital staff and appreciated the growing number of clients referred from other medical departments.

Developing a clear sense of professional identity resulted in a self-sustainable OT department with an established position within the hospital. The practitioners realised their contribution to society because TV broadcasts covered OT, health insurances covered OT, and even clients paid out-of-pocket [31, 33, 36]. Eventually, the OT department was handed over to local staff taking full responsibility. Few previous studies focused on professional identity in the context of OT in cross-cultural settings. Lavine and Greiner regarded the image of OT as a critical aspect fostering the implementation of OT services in China [39]. Several authors consider self-identity a relevant aspect of learning experiences [6, 81, 87]. Professionalization and a clear understanding of OT within society are essential factors for the successful implementation of OT [85, 88].

### 4.3. Cultural Awareness in Expansion

During implementation, practitioners realised that their clients are from various resident nationalities, but they regarded cultural differences as not important (“Culture does not matter, we treat all the same”). The OTs wanted to treat everybody “the same,” without any cultural prejudice [44]. The different languages were perceived as treatment barriers, and conversations about religion were not initiated. The therapists expressed that the level of education matters more than the cultural background for the receptiveness of therapy. Over time, the OTs realised that they adapted their treatment by addressing the different cultural backgrounds, health understandings, and literacy [20]. They described what culture means for them and developed their definition of culture. Indeed, repetitive engagement with diverse nationalities stimulates cultural awareness and competences [19–22]. Over time, the OTs developed a basic understanding of the other languages (consistent with the findings of Alizadeh and Cavan [19]) and requested translators more frequently [89]. The practitioners understood that a sensitive cross-cultural practice is only achieved by an ongoing and intentional process of cultural learning, thus developing cross-cultural understanding [21, 22]. The OT's recognised that the religious faith of the clients is part of their culture and should be acknowledged and respected. The practitioners explored the values and beliefs of their clients without imposing their own beliefs on others [20]. They recognised the uniqueness, dignity, and potential of individuals within their social and cultural background [21]. The practitioners realised that mutual cultural learning improved their competencies as OT. Our findings support current concepts of OT in cross-cultural contexts [17, 19, 72]. OT theory and practice built on the diverse cultural background of clients and practitioners can have a relevant and significant impact [1, 20–22, 81]. OT implemented in diverse settings around the world, and the benefits of sensitive cross-cultural practice were observed consistently [12, 16, 72]. It needs an understanding of local beliefs, values, and meaning of occupation [12, 86]. If engaged with mutual understanding and respect, cross-cultural experiences have enriching mutual benefits [9, 17, 19, 20, 90]. Culturally responsive care in OT is a valuable tool to address the future needs of a global community [8, 91]. It appears that culturally sensitive OT affects patient's health by considering habits, routines, family, and community culture within their daily life context [81].

### 4.4. Methodological Considerations

The strength of our study is that we observed the local OTs two times in a period of eight years. This approach helped to comprehensively document the changing perspectives in the course of establishing the department. Besides, the study was conducted in a unique setting with an incredibly diverse non-Western population. Also, the interviews were carried out sensitively, aiming to represent as closely as possible the voices of those under study. Several limitations could be discussed: First, only few participants were studied. However, all staff working at the department participated at the respective time points, making a selection effect unlikely. Second, the results represent the situation at a specific time point in a unique setting, which cannot be applied to other situations and settings. However, our findings are essentially in line with previous studies, thus confirming our interpretation. Finally, the number of researchers conducting interviews and interpreting the answers was limited, possibly affecting the findings and interpretations.

## 5. Conclusions

The present research reports the development and successful implementation of an OT department in a unique Himalayan cross-cultural setting. Local rehabilitation therapists were empowered in a long-term hands-on apprenticeship model, and responsibility was handed over step by step. The OTs were eventually able to practice and explain professional content with confidence. They recognised OT interventions as a process of change, actively involving the clients and raising hopes and perspectives in clients and families. The practitioners developed a clear sense of professional identity and their role within the hospital and society. They became aware of their skills and abilities and were able to explain the broader impact of OT services to clients, medical staff, friends, and family. The OTs recognised that daily life habits, routines, families, and community culture affect a patient's health and that a culturally sensitive OT practice can have a significant impact. More and more, the practitioners adapted their treatment by addressing the different cultural backgrounds, languages, health understandings, and education. They understood that a sensitive cross-cultural practice is only achieved by an ongoing and intentional process of cultural learning, thus developing cross-cultural understanding. It appears that the concepts and principles of OT can be applied in non-Western cross-cultural settings. OT services helped to meet the urgent need for rehabilitation in China which emerges after the vast earthquake in 2010. These findings support the vision of the WFOT to “enable all people regardless of culture, ethnicity, or social background, to be engaged in occupations that support their well-being and the well-being of their communities.”

## Figures and Tables

**Table 1:** Interview guide.

Your work
*1. Can you tell me about your daily work at “Qinghai Red Cross Hospital”?*
*2. How do you explain your work to a friend?*
*3. How do you introduce your work to a client?*
Professional identity and position within the QHRCH hospital
*4. How is your position as an OT in your department within the hospital?*
*5. How is your position as an OT within the hospital?*
*6. Doctors have the best understanding about what you can do?*
*7. How are your possibilities for professional development?*
Who are your clients?
*8. Can you tell me about some of your clientele? Can you give some examples of their diagnoses?*
Client's understanding of OT?
*9. How do clients know about the OT service? What kind of treatment do they expect if they are coming to see you?*
*10. Are clients willing to pay for therapy? Which treatment sells best?*
What are your therapy aims? What do you do practically as OT?
*11. Could you tell me something about your therapy approach, what do you do with the client? What kind of activities you carry out during therapy?*
Does culture influence your work or the relationship with the client?
*12. What is your definition of culture? What is it?*
*13. Do cultural backgrounds influence your therapy work? Do you treat patients from different nationalities differently? Any specifics?*
*14. Do you adapt your treatment because of the cultural background of a client? Specific example?*
Other aspects for treatments
*15. How do you perceive communication with people from resident nationalities? Can you understand each other? What languages do you speak?*
*16. What do you think about science and evidence-based practice? Do they matter for your practice? If yes, how and what kind of?*
Specifics about your own cultural learning
*17. Could you describe a situation in which cultural learning occurred for you?*
*18. What part of that experience would you consider cultural learning?*
End of the interview
*19. Do you have anything more to say or anything you would like to add?*
*20. Do you have more comments concerning your work, your professional identity, and how culture influences how you work?*

**Table 2:** Participants' characteristics at the time of the second field study 2017, including years of practice at QHRCH and participation in study 1 and study 2.

Participant No.	Gender	Age	Years of practice	Field 2009	Field 2017
1	M	31	8	x	x
2	F	29	6	x	x
3	F	32	7	x	x
4	F	28	5		x
5	M	27	4		x
6	F	26	3		x
7	M	31	2		x

## Data Availability

The interview guide, table of participant characteristics, and mathematical framework of the findings are included within the article.

## Additional Points

*Authorship Details.* The following are the authorship details: Gaby Scheidegger, ErgoService, 3270 Aarberg, Switzerland, praxis@ergoservice.net.
